# Inguinal Lymphadenectomy for Penile Cancer: An Interim Report from a Trial Comparing Open Versus Videoendoscopic Surgery Using a Within-patient Design

**DOI:** 10.1016/j.euros.2024.02.007

**Published:** 2024-03-21

**Authors:** Marco Falcone, Murat Gül, Federica Peretti, Mirko Preto, Lorenzo Cirigliano, Martina Scavone, Omid Sedigh, Marco Oderda, Paolo Gontero

**Affiliations:** a Urology Clinic, A.O.U. Città della Salute e della Scienza, Molinette Hospital, University of Turin, Turin, Italy; b Neurourology Clinic, Unità Spinale Unipolare, A.O.U. Città della Salute e della Scienza, Turin, Italy; c Department of Urology, Selcuk University School of Medicine, Konya, Turkey; d Urological Department, Gradenigo Hospital, Turin, Italy

**Keywords:** Penile cancer, Inguinal lymphadenectomy, Open surgery, Video laparoscopic surgery

## Abstract

**Background and objective:**

Nodal metastasis is a major survival and prognostic factor in penile cancer (PeCa). Thus, accurate staging, prognosis, and treatment selection require adequate inguinal lymphadenectomy (ILND). ILND surgery should balance oncologic rigor with morbidity and postoperative complications. Our aim was to compare the feasibility and safety of open ILND (OILND) and videoendoscopic ILND (VEILND) in patients with PeCa.

**Methods:**

We conducted a single-center randomized trial with a within-patient design between October 2019 and April 2023. Patients who were undergoing either staging or radical ILND for PeCa were included and randomized to receive either OILND or VEILND on one side, with the other technique then used on the contralateral side. The trial was approved by the local ethics committee and was registered on ClinicalTrials.gov (NCT05887921). The primary outcome was the safety of VEILND. Secondary outcomes included intraoperative and postoperative morbidity rates and surgical outcomes for the two procedures, as well as oncological outcomes according to survival estimates.

**Key findings and limitations:**

We included 14 patients in the study. Median follow-up was 12 mo (interquartile range [IQR] 12–17). There were no significant differences in operative time and the number of lymph nodes removed between OILND and VEILND. However, the median time to drain removal was significantly shorter in the VEILND group (15 d, IQR 13–17, 95% confidence interval [CI] 12–17) than in the OILND group (27 d, IQR 20–41, 95% CI 24–31; *p* = 0.025). No intraoperative complications were observed, but postoperative complications occurred in three cases (21.4%, 95% CI 8.4–37.8%) in the VEILND group and eight (57.1%, 95% CI 18.6–54.3%) in the OILND group (*p* = 0.032).

**Conclusions and clinical implications:**

VEILND represents a safe technique to consider for either staging or curative intent in PeCa and seems to have an advantage over OILND in terms of morbidity. Further high-powered studies are warranted to confirm these preliminary results.

**Patient summary:**

We compared the outcomes of two different surgical techniques to remove lymph nodes in patients with penile cancer. We found that a video-assisted keyhole surgery approach seems to result in a lower rate of complications than after open surgery.

## Take Home Message

Radical resection of inguinal lymph nodes using a videoendoscopic technique is a feasible option in patients with penile cancer.

## 1 Introduction

In Western countries, penile cancer (PeCa) accounts for <1% of all male malignancies [1]. Nevertheless, it remains a significant public health concern because of its aggressive nature and poor prognosis, especially in advanced stages. Standard treatment for localized PeCa consists of surgical removal of the primary tumor, followed by inguinal lymphadenectomy (ILND) for patients with either high-risk disease or lymph node involvement [2], [3].

In PeCa, nodal metastasis is a significant prognostic factor and one of the most influential survival factors. Consequently, adequate ILND is essential for accurate staging, prognosis, and selection of appropriate treatment strategies. The surgical approach chosen for ILND should strike a balance between the need for oncological rigor and the risk of morbidity, particularly concerning the incidence of postoperative complications [2], [3]. Open ILND (OILND) and videoendoscopic (VEILND), either simple or robot-assisted (R-VEILND), are the surgical approaches most frequently used. OILND is associated with considerable morbidity because of impaired lymph drainage from the legs and scrotum, with the incidence of complications reported ranging from 21% to 55% [4], [5]. VEILND is considered a minimally invasive option that may reduce postoperative complications [6], [7]. Contemporary data indicate comparable short-term outcomes, although these results are mostly based from nonrandomized trials [8], [9], [10].

The aim of the current study was to compare surgical outcomes for OILND and VEILND in a prospective randomized study in patients with PeCa.

## 2 Patients and methods

This randomized single-center trial was approved by the local ethics committee (protocol no. 0036656) and registered on ClinicalTrials.gov (NCT05887921). Before study participation, all patients signed an informed consent form.

### 2.1 Eligibility criteria

This clinical trial enrolled patients undergoing either staging or radical ILND for PeCa. Inclusion criteria for the participants were age >18 yr and an indication for lymphadenectomy for PeCa cancer according to the European Association of Urology (EAU) guidelines [2]. The exclusion criteria were age >80 yr, Charlson comorbidity index ≥3, diagnosis of a lymphatic disorder of the lower limbs, anamnesis of previous inguinal surgery/radiotherapy and/or a prior or concurrent diagnosis of another malignancy. The study patients were randomized to receive OILND or VEILND in one groin, and then the other technique on the contralateral side, using a computerized random selection generator.

### 2.2 Surgical technique for ILND and VEILND

**Fig. 1:**
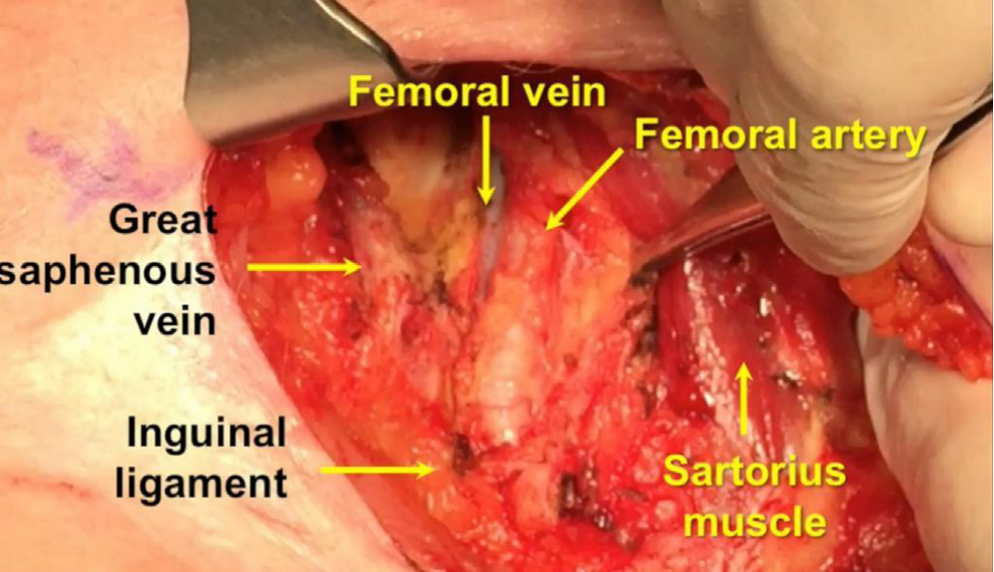
Limits of Scarpa’s triangle in open inguinal lymph node dissection: the upper limit is the inguinal ligament, the lateral limit is the sartorius muscle, and the medial limit is the adductor muscle. Excision of the lymph nodes proceeds along the course of the femoral vessels and the saphenous vein, which is spared if possible.

OILND procedures were performed by surgeons experienced in the management of PeCa (M.F. and O.S.). A fascia-sparing ILND (fs-ILND) procedure was performed in all cases (Fig. 1).

The surgical procedure included the following steps:

­ A transversal inguinal incision of approximately 7 cm in length was made 1 cm below the inguinal ligament.
­ Camper’s fascia was incised.
­ Superficial nodal stations between Camper’s fascia and the lata fascia were dissected.
­ The saphenous veins were isolated and spared if not involved in the diseased nodal package.
­ Progressive ligation of the pudendal, epigastric, and circumflex vascular pedicles was performed.
­ Superficial lymph-node packages were isolated.
­ Deep nodal packages were isolated and excised at the level of the ovalis fossa.
­ Lymphostasis was achieved via selective use of metallic ligating clips and application of an automatic hybrid (ultrasound/bipolar) sealing system (Thunderbeat; Olympus Medical Systems, Tokyo, Japan)
­ Hemostasis was achieved and a tubular drain was left in place.

To maximize the minimally invasive nature of the procedure, we tried to preserve the saphenous vein (if surgically possible) and never used a pedicled sartorius muscle flap to cover the dissected inguinal area.

**Fig. 2:**
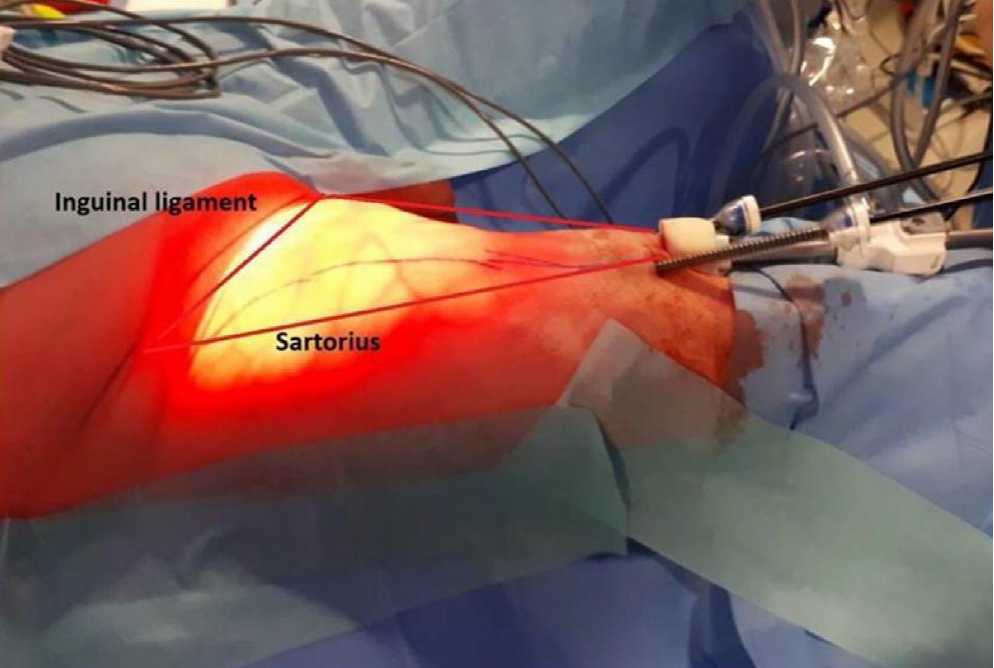
Intraoperative appearance of videoendoscopic inguinal lymph node dissection with details of groin superficial vascularization. Laparoscopic dissection proceeds up to the inguinal ligament.
­ The pudendal, epigastric, and circumflex pedicles were isolated and selectively ligated using ligating clips or an automatic hybrid (ultrasound/bipolar) sealing system (Thunderbeat).
­ The saphenous vein was isolated and spared if possible.
­ The superficial lymph-node packages were excised.
­ Deep lymph-node packages were excised at the level of the ovalis fossa.
­ After hemostasis, a tubular drain was left in place.

The surgeons who performed VEILND (M.O. and M.F.) had good expertise in laparoscopic techniques and were familiar with OILND. The VEILND procedure comprised the following steps:

­ A Hasson trocar was inserted at the apex of Scarpa’s triangle.
­ A working space below Camper’s fascia was digitally developed.
­ Two 5-mm trocars were placed 5 cm laterally to the Hasson trocar in a triangular fashion.
­ Progressive development of the working space was achieved via CO_2_ insufflation at 10 mm Hg (Fig. 2).

**Fig. 3:**
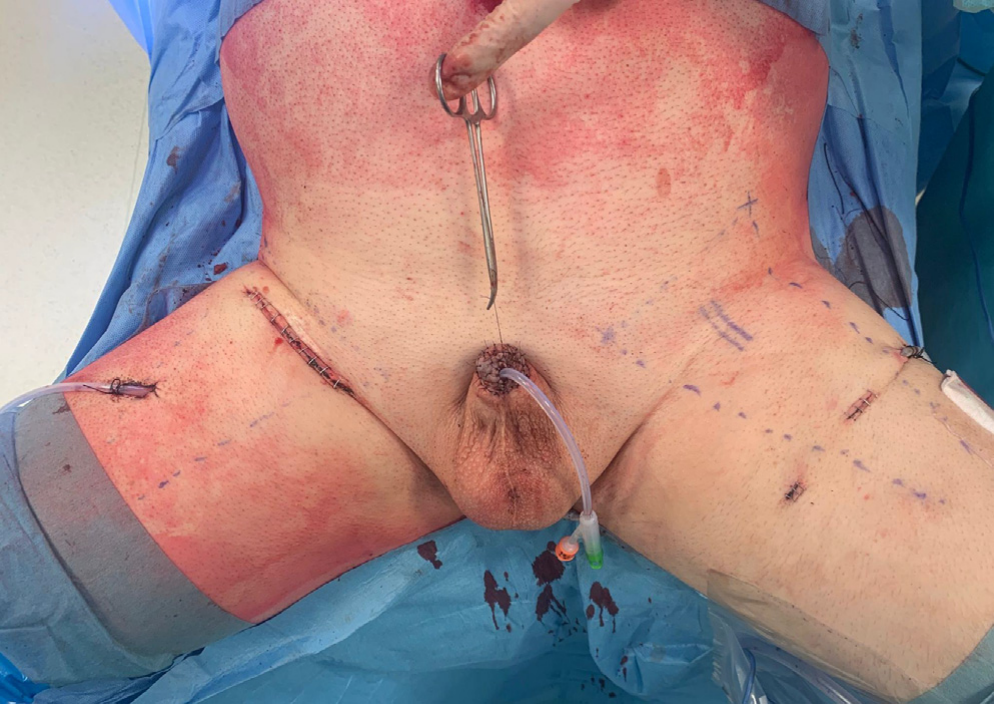
Postoperative appearance after open inguinal lymph node dissection (ILND) in the right groin versus videoendoscopic ILND in the left groin.

The final appearance after OILND and VEILND is shown in Figure 3.

### 2.3 Outcome measures and follow-up evaluation

Preoperative patient characteristics included median age (years), smoking habit, and body mass index. Details for clinical lymph node staging, pathological stage and grade of primary the penile tumor (TNM), and the indication for ILND (staging or radical treatment) were recorded. The intraoperative parameters analyzed were the operative time, saphenous preservation, and the number of nodes excised and of positive nodes.

Postoperative data collected consisted of the median 5-d drainage volume, time to drain removal, and median length of hospital stay. After their operation, patients attended a 24-mo follow-up program in accordance with the EAU guidelines [11]. Postoperative complications (including early thigh lymphedema and lymphocele, and 3-mo thigh lymphedema and leg discomfort) were recorded. Postoperative pain was evaluated using a validated questionnaire (Visual Analog Scale score) on postoperative day 1 [12]. Oncological outcomes were analyzed in terms of the 1-yr recurrence-free and cancer-specific survival rates.

### 2.4 Study endpoints

The primary endpoint was the safety of VEILND. Secondary endpoints included intraoperative and postoperative complications and surgical outcomes (number of surgically removed lymph nodes and of positive nodes) for VEILND versus OILND. We also evaluated the 1-yr recurrence-free and cancer-specific survival rates.

### 2.5 Randomization method

A within-patient randomized design with allocation of patients at a 1:1 ratio to either the OILND group or the VEILND group was applied. The Department of Biostatistics at Turin University Hospital generated randomized, sequentially numbered, opaque sealed envelopes. A simple randomization system with application of a computer-generated (SPSS version 28.0.1.0; IBM, Armonk, NY, USA) set of random numbers was used. A research nurse opened the envelopes consecutively after each patient was registered for the trial and each groin was then assigned to either the OILND or the VEILND group.

### 2.6 Statistical analysis

SPSS version 28.0.1.0 was used for statistical analysis. Categorical variables are reported as the frequency and percentage, and continuous variables as the median and interquartile range (IQR). Differences between groups were assessed using a Wilcoxon signed-rank test or McNemar’s test (for binary data) on the basis of the data distribution. The Kaplan-Meier method was used to estimate recurrence-free and cancer-specific survival rates. Missing values were imputed using the mean. Two-sided *p* values <0.05 were considered to be statistically significant.

## 3 Results

### 3.1 Study population and baseline characteristics

A total of 17 patients were eligible for the study between October 2019 and April 2023. Of these, three patients were excluded: one was unlikely to benefit from surgery because of metastatic disease, and the other two were unfit for surgery. Therefore, data for 28 randomized groins were included in the trial. Baseline characteristics of the study population are listed in Table 1. All patients underwent ILND (performed as OILND on one side and VEILND on the other side in each patient) and completed at least 12-mo follow-up. Median follow-up was 14 mo (IQR 12–17).

**Table 1:** Preoperative characteristics of patients who underwent ILND

Parameter	Result
Median age, yr (IQR)	63 (57–69)
Current smoker, n (%)	4 (14.3)
Median BMI, kg/m^2^ (IQR)	29 (26–32)
cN stage, *n* (%)	
cN0	21 (75)
cN1	5 (17.9)
cN2	2 (7.1)
T stage, *n* (%)	
*T2*	22 (78.6)
*T3*	6 (21.4)
Grade, *n* (%)	
Grade 2	19 (67.9)
Grade 3	9 (32.1)
Positive margins, *n* (%)	2 (7.1)
ILND indication, *n* (%)	
Staging	20 (71.4)
Radical treatment	8 (28.6)

BMI = body mass index; ILND = inguinal lymph node dissection; IQR = interquartile range.

### 3.2 Intraoperative and postoperative outcomes

No intraoperative complications occurred during the procedures. No statistically significant differences were found for the operative time, number of lymph nodes removed, or number of positive nodes (Table 2). Time to drain removal was significantly longer in the OILND group (27 d, IQR 20–41, 95% confidence interval [CI] 24–31) than in the VEILND group (15 d, IQR 13–17, 95% CI 12–17; *p* = 0.025; Table 3). Although the difference did not reach statistical significance, the median 5-d drain volume was greater in the OILND group (75 ml, IQR 35–150, 95% CI 40–92) than in the VEILND group (50 ml, IQR 40–73, 95% CI 33–67; difference 25 ml; *p* = 0.131).

**Table 2:** Intraoperative parameters for patients who underwent ILND

Parameter	Total	OILND	VEILND	OR (95% CI*)*	*p* value a
Groins (*n*)	28	14	14		
Median operative time, min (IQR)	92 (90–128)	93 (74–123)	93 (90–130)	–	0.638
Saphenous vein preservation, *n* (%)	26 (92.9)	12 (85.7)	14 (100)	NC	0.338
Intraoperative complications, *n* (%)	0 (0)	0 (0)	0 (0)	–	1.0
Median no. of LNs excised (IQR)	8 (6–13)	8 (7–14)	8 (6–13)	–	0.55
Median no. of positive LNs (IQR)	0 (0–0)	0 (0–0)	1 (0–0)	–	0.63
pN+ groin, *n* (%)	5 (17.8)	3 (21.4)	2 (14.2)	1.64 (0.422–6.448)	0.516

CI = confidence interval; ILND = inguinal lymph node dissection; IQR = interquartile range; LNs = lymph nodes; NC = not calculable; OILND = open ILND; OR = odds ratio; VEILND = videoendoscopic ILND.

a The Wilcoxon signed-rank test was used to determine significance.

**Table 3:** Postoperative data for patients who underwent ILND

Parameter	Overall	OILND (*n* = 14 groins)		VEILND (*n* = 14 groins)		OR (95% CI) a	*p* value b
		Result	95% CI	Result	95% CI		
Median time to drain removal, d (IQR)	20 (14–30)	27 (20–41)	24–31	15 (13–17)	12–17		**0.025**
Median 5-d drain discharge, ml (IQR)	59 (40–88)	75 (35–150)	40–92	50 (40–73)	33–67		0.131
Median length of hospital stay, d (IQR)	5 (4–9)	–		–			
Postoperative complications, *n* (%)	11 (39.3)	8 (57.1)	18.6–54.3%	3 (21.4)	8.4–37.8%	2.44 (0.464–12.86)	**0.028**^d^
Early thigh lymphedema, *n* (%)	6 (21.4)	4 (28.6)	13.2–48.7%	2 (14.3)	4.3–24.4%	2.4 (0.365–15.9)	0.69 d
Lymphocele, *n* (%)	2 (7.1)	1 (7.1)	0.2–33.6%	1 (7.1)	0.2–33.6%	1 95 (0.130–7.63)	1 d
Median VAS at postoperative day 1 (IQR)	2 (2–3)	3 (2–4)	1.3–4.7	2 (2–3)	1.1–2.9		0.428
Thigh lymphedema at 3 mo, *n* (%)	5 (17.9)	5 (35.7)		0 (0)		– c	**0.016**^d^
Leg discomfort at 3 mo, *n* (%)	4 (14.3)	4 (28.6)		0 (0)		– c	**0.019**^d^
Recurrence-free survival at 1 yr, *n* (%)	13 (92.8)	–		–			
Cancer-specific survival at 1 yr, *n* (%)	13 (92.8)	–		–			
Median follow-up, mo (IQR)	14 (12–17)	–		–			

CI = confidence interval; ILND = inguinal lymph node dissection; IQR = interquartile range; OILND = open ILND; OR = odds ratio; VAS = Visual Analog Scale score for pain; VEILND = videoendoscopic ILND.

a The OR and 95% CI were computed using the OILND data set as the reference.

b The Wilcoxon signed-rank test was used to determine significance unless otherwise indicated.

c The OR could not be calculated for the VEILND group as no complications were observed in this cohort.

d McNemar’s test was used to determine significance.

The frequency of postoperative complications was significantly lower in the VEILND group than in the OILND group (OR 3.16, 95% CI 0.464–12.86; *p* = 0.028). Specifically, at 3 mo after surgery, no patients in the VEILND group (0%) had experienced thigh lymphedema, compared to 35.7% of patients in the OILND group (*p* = 0.016). Leg discomfort at 3 mo after ILND was also significantly more frequent in the OILND group (28.6%) than in the VEILND group (0%; *p* = 0.019). We did not observe any cases of skin necrosis in either group.

### 3.3 Oncological outcomes

During the clinical trial, only two patients experienced systemic recurrence and died of PeCa. Overall, the 1-yr recurrence-free survival rate was 92.8% and the 1-yr cancer-specific survival rate was 92.8%.

## 4 Discussion

Appropriate management of regional lymph nodes is critical for the survival of patients suffering from PeCa. The most crucial factor in predicting outcomes for these patients is the presence and extent of regional nodal involvement [11]. Limited disease restricted to the regional lymph nodes can be curable, for which the preferred treatment option is radical ILND, especially for patients with cN1–2 disease [11]. Nonetheless, ILND is still considered an alternative option to sentinel node biopsy for regional nodal staging of intermediate- or high-risk PeCa [13].

We conducted a randomized comparative analysis of ILND outcomes in PeCa using two distinct techniques: OILND and VEILND. Our findings indicate that VEILND has multiple advantages over OILND, specifically in relation to the occurrence of postoperative complications.

Studies and reviews in the literature have demonstrated the efficacy of ILND performed using the traditional open technique in achieving oncological control [14]. However, this approach is associated with a notable incidence of postoperative complications, such as skin infection, surgical wound dehiscence, and lymphocele [15]. After the first cadaveric endoscopic ILND was reported by Bishoff et al [16] in 2003, growing interest and expertise in minimally invasive approaches have resulted in the emergence of VEILND and then robot-assisted VEILND [17], [18], [19], [20], [21], [22], [23].

To date, only a few high-powered studies have compared the different surgical approaches for ILND. In 2006, a prospective randomized study conducted in collaboration with the Brazilian National Institute of Cancer compared OILND in one limb with VEILND in the contralateral limb within the same patient, thereby minimizing potential biases arising from interpatient variations [24]. A total of ten patients with impalpable nodes underwent bilateral ILND. The study authors observed that VEILND resulted in a significant reduction in morbidity in comparison to OILND (20% vs 70%; *p* = 0.059). Remarkably, despite the lower morbidity, the same number of lymph nodes was removed with VEILND as with OILND [24].

Tobias-Machado et al [6] reported on prospective follow-up for 15 consecutive patients who underwent VEILND for PeCa. In the initial phase, bilateral inguinal dissections were performed in ten patients, with VEILND performed on one side and OILND on the other side for nonpalpable lymph nodes. There were no significant differences in the number of nodes removed or of positive nodes between the two techniques. However, complications were observed in 70% of limbs in the OILND group, compared to only 20% of limbs in the VEILND group (*p* = 0.015). Furthermore, patients who underwent bilateral VEILND were able to be discharged from the hospital within an average of 24 h (range 12–36 h), while those who underwent OILND in addition to contralateral VEILND had an average hospital stay of 6.4 d (range 5–10 d) [6].

Kumar and Sethia [20] reported outcomes for 42 consecutive patients who underwent ILND for PeCa between 2008 and 2015. During the study period, a total of 68 ILND procedures were performed, of which 35 were OILND and 33 were VEILND. No intraoperative complications were observed in either group. However, incidence rates for wound complications and leg lymphocele were significantly lower in the VEILND group than in the OILND group (both *p* < 0.001). Importantly, comparable lymph node yield, mean number of positive lymph nodes, and lymph node density were observed in the VEILND group, confirming its oncological safety and efficacy. In addition, VEILND resulted in a significant reduction in the mean length of hospital stay, with a difference of 4.8 d (*p* < 0.001), in comparison to OILND [20].

A prospective randomized study by Yadav et al [25] enrolled 29 patients requiring ILND for PeCa. Participants were randomly assigned to undergo either VEILND or OILND, with the right and left groins representing the respective surgical approaches. The study revealed that although the mean operative time was significantly longer for VEILND than for OILND (162.83 vs 92.35 min; *p* < 0.001), median hospital stay and time to return to usual activities were both shorter in the VEILND group (both *p* < 0.01). The mean number of lymph nodes removed was 7.6 in the VEILND group and 8.3 in the OILND group, representing a similar lymph node yield (*p* = 0.681). Postoperative complications were observed in ten limbs (34.48%) in the OILND group and three limbs (10.34%) in the VEILND group. Specifically, while there was no significant difference observed for lymphedema or lymph collection, wound infection (*p* < 0.01) and skin necrosis (*p* = 0.01) were significantly more frequent in the OILND group [26]. Taken together, findings from all of these studies support the advantages of VEILND in terms of lower wound complication rates, comparable oncological outcomes, and significantly shorter hospital stays, highlighting its potential as a preferred approach over OILND in PeCa.

A 2022 meta-analysis by Patel et al [9] included two randomized controlled trials (RCTs) and six cohort studies. The findings from their comprehensive analysis align closely with the conclusions drawn from our study, indicating that VEILND is associated with a lower incidence of skin and lymphatic complications, specifically wound infection, dehiscence, and lymphedema. Furthermore, endoscopic approaches are associated with shorter hospital stays and earlier removal of groin drains. Notably, an emerging trend observed in these studies is that minimally invasive ILND techniques consistently result in retrieval of a greater number of lymph nodes than with the open technique. These findings collectively emphasize the advantages of endoscopic techniques for lymphadenectomy in terms of better surgical outcomes and enhanced patient recovery.

Our study aligns with the existing body of evidence, reinforcing the findings observed in previous comparative studies and meta-analyses. Specifically, VEILND was associated with earlier drain removal (*p* = 0.012) and a lower median drain discharge volume (*p* = 0.329) in comparison to OILND. In addition, VEILND was associated with a significantly lower rate of postoperative complications, including lower rates of lymphedema (*p* = 0.04) and leg discomfort (*p* = 0.028) at 3 mo. These results add to the evidence base in favor of the videoendoscopic approach, with more selective identification of lymphatic branches and more efficient lymphostasis. However, in our study, in contrast to some studies in the literature, the number of lymph nodes retrieved and positive nodes did not significantly differ between the two groups, indicating similar oncological outcomes could be obtained via both techniques. These data could be biased by both the wide experience of surgeons performing OILND and the need to complete the full learning curve by the team performing VEILND.

There are many surgical templates to consider for patients with intermediate- or high-risk cN0 disease, including modified ILND, superficial fs-ILND, and classical rILND, and the template applied may strongly affect the number of nodes excised and consequently possible surgical outcomes [26], [27]. In the current study we were limited to evaluating surgical outcomes of superficial fs-ILND using an open versus a minimally invasive surgical approach.

Our study has several strengths. First, as a comparative study it allowed direct assessment of the advantages and disadvantages of each approach. Second, different from other studies in which OILND was used as a comparator [11], we avoided some morbidity-associated steps such as sartorius transposition and saphenous vein sacrifice. The omission of these steps allows a more direct comparison of the main surgical techniques being evaluated, with a focus on the differences between OILND and VEILND without the confounding effects of additional invasive steps. Nevertheless, it is crucial to recognize and address the limitations to our study. Although the study used a randomized design, we performed both techniques in the same patient in all cases. This design allowed us to minimize any bias due to interpatient variations, however, we could not compare some postoperative outcomes (eg, length of hospital stay) and oncological outcomes (eg, recurrence-free and cancer-free survival rates) between the techniques. To strengthen the evidence, a prospective RCT including bilateral procedures is necessary. Results from the VELRAD RCT comparing OILND to VEILND are awaited, which includes patients who require lymphadenectomy for PeCa, urethral cancer, scrotal cancer, or melanoma, with no contraindications to surgical intervention for their cancer [28].

One of the limitations of our study is that the surgeons involved differed in both age and surgical experience, so the different learning curves may have influenced surgical performance and consequently surgical outcomes. Another factor that may be a source of possible bias is the lymphadenectomy surgical template applied (superficial vs radical). Other limitations are the relatively small sample size and short follow-up, which may limit the generalizability of our findings, particularly for oncological outcomes. The lack of node density assessment as an outcome measure is an additional limitation.

Despite several limitations, given the rare incidence of PeCa and the paucity of data comparing the two surgical techniques, we believe that our data add to the current body of evidence. Further results for a larger sample with longer follow-up will allow more comprehensive evaluation of the potential advantages of VEILND over OILND.

## 5 Conclusions

VEILND represents a safe technique to consider for either staging or curative intent in PeCa. VEILND seems to have an advantage over OILND in term of morbidity. Progression of the surgical learning curve for VEILND may improve surgical outcomes in the future and further high-powered studies that include bilateral procedures and longer-term follow-up are warranted to confirm our preliminary results.

  ***Author contributions*:** Marco Falcone had full access to all the data in the study and takes responsibility for the integrity of the data and the accuracy of the data analysis.

  *Study concept and design:* Preto, Falcone, Sedigh.

*Acquisition of data:* Peretti, Cirigliano, Scavone, Oderda.

*Analysis and interpretation of data:* Peretti, Falcone.

*Drafting of the manuscript:* Peretti, Gül.

*Critical revision of the manuscript for important intellectual content:* Falcone, Gül, Gontero.

*Statistical analysis:* Gül, Falcone.

*Obtaining funding:* None.

*Administrative, technical, or material support:* None.

*Supervision:* Gontero.

*Other:* None.

  ***Financial disclosures*:** Marco Falcone certifies that all conflicts of interest, including specific financial interests and relationships and affiliations relevant to the subject matter or materials discussed in the manuscript (eg, employment/affiliation, grants or funding, consultancies, honoraria, stock ownership or options, expert testimony, royalties, or patents filed, received, or pending), are the following**:** Marco Falcone and Murat Gül are members of the European Association of Urology Sexual and Reproductive Health Guidelines Panel. The remaining authors have nothing to disclose.

  ***Funding/Support and role of the sponsor*:** None.
